# Video-based cognitive-behavioral intervention for COVID-19 anxiety: a randomized controlled trial

**DOI:** 10.47626/2237-6089-2020-0056

**Published:** 2021-05-08

**Authors:** Reza Shabahang, Mara S. Aruguete, Lynn McCutcheon

**Affiliations:** 1 University of TehranTehranIran University of Tehran, Tehran, Iran.; 2 Lincoln UniversityJefferson CityMOUSA Lincoln University, Jefferson City, MO, USA.; 3 North American Journal of PsychologyWinter GardenFLUSA North American Journal of Psychology, Winter Garden, FL, USA.

**Keywords:** COVID-19, video-based psychotherapy, cognitive-behavioral therapy, anxiety

## Abstract

**Objective:**

Cognitive-behavioral interventions can be effective for relieving anxiety associated with coronavirus disease 2019 (COVID-19), but complications such as social distancing, quarantine, a shortage of experts, and delayed care provisions have made it difficult to access face-to-face therapeutic interventions. The purpose of this study was to investigate the efficacy of a video-based cognitive-behavioral intervention for reducing COVID-19 anxiety.

**Method:**

In the present randomized controlled trial, 150 college students with severe COVID-19 anxiety were randomly assigned to either an intervention (n = 75) or a waiting list control (n = 75) group. The intervention group participated in a video-based cognitive-behavioral program consisting of nine 15-20-minute sessions (three days a week for three weeks). Dependent measures included the COVID-19 Anxiety Questionnaire, Short Health Anxiety Inventory, Anxiety Sensitivity Index-3, Somatosensory Amplification Scale, Experience of Parasocial Interaction Scale, and Source Credibility Scale.

**Results:**

Participants who were randomly assigned to the cognitive-behavioral program reported high parasocial interaction, source credibility, and satisfaction with the intervention. Eighty percent reported that the video-based intervention was a beneficial alternative to traditional face-to-face therapeutic intervention. At post-treatment evaluation, the video-based cognitive-behavioral intervention group showed a significant reduction in COVID-19 anxiety, health anxiety, anxiety sensitivity, and somatosensory amplification when compared to the wait-listed control group.

**Conclusions:**

This study suggests that video-based cognitive-behavioral interventions can be an affordable, feasible, and effective method to reduce anxiety during a large-scale pandemic.

## Introduction

Health anxiety, or distress related to fears of contracting a disease, is a widespread problem associated with a range of psychological and behavioral symptoms.^1^ While health anxiety is already common, there is evidence that such distress is intensified and more widespread during public health crises, such as the Ebola^2^ and coronavirus disease 2019 (COVID-19) outbreaks.^3,4^ Cognitive-behavioral therapies show excellent efficacy for the treatment of health anxiety.^5^ However, during a public health crisis, it may be difficult to access traditional therapy due to a number of barriers, including the need for social distancing to prevent disease transmission, a lack of therapists to meet increased mental health needs, financial limitations, and stigma over seeking mental health treatment. Cognitive-behavioral video-based interventions can potentially alleviate the increased mental health needs during public health crises. The present study tests the efficacy of a cognitive-behavioral video-based intervention program for health anxiety during the COVID-19 epidemic in Iran.

Health anxiety is characterized by a preoccupation with physiological cues or “symptoms” that leads some to believe that they are suffering from, or will acquire, a serious illness.^1^ Health anxiety may be manifested in health-related behaviors such as excessive investigation into health conditions, maladaptive avoidance of situations or substances deemed unhealthy, and frequent and unnecessary visits to health care facilities. Individuals with health anxiety often persist in the belief that they have a serious illness despite medical assurance of the opposite.^1,6^ Indeed, health anxiety results in significant costs due to unnecessary use of medical services,^7^ which can result in financial hardship. In addition, those who suffer from health anxiety often experience occupational and social problems^8^ and generally low quality of life.^9^ Therefore, the costs of health anxiety extend well beyond immediate psychological distress.

Health anxiety may be amplified in large populations during disease outbreaks. There is evidence that diseases such as Ebola and COVID-19 are associated with a widespread collective fear that is out of proportion to the actual physical threat for many people.^2,10^ For example, a poll recently conducted by the American Psychiatric Association found that 40% of the Americans report anxiety over dying or becoming seriously ill from COVID-19, despite the fact that the vast majority of cases only show mild symptoms.^11^ Sleep difficulties, fears of contagion, and social media stress have been common during the COVID-19 pandemic, and 80% of respondents in another study indicated the need for increased mental health care during the pandemic.^3^ Media exposure to coverage of disease outbreaks was shown to be an important determinant of health anxiety during the Ebola epidemic^2^ and also in the recent COVID-19 pandemic.^4^ Indeed, about half of the participants in one study reported feeling panic after hearing news reports about COVID-19.^3^ Media coverage may cause anxiety, yet it also plays an important role in alerting populations to the seriousness of disease outbreaks and educating them about prevention strategies. Therefore, it is important to find ways of managing anxiety during large-scale disease outbreaks.

Anxiety sensitivity and somatosensory amplification contribute to health anxiety.^12,13^ Anxiety sensitivity is characterized by fear of arousal-related sensations^14^ and is considered a risk factor for health anxiety in cognitive-behavioral models of health anxiety.^12^ Furthermore, somatosensory amplification, or the tendency to experience normal somatic and visceral sensations as intense, noxious, and disturbing,^15^ plays a prominent role in the development of health anxiety.^13^ There is evidence that communities have experienced elevated levels of anxiety sensitivity and somatosensory amplification during the COVID-19 outbreak. These symptoms are likely to contribute to widespread health anxiety in response to COVID-19.^16-18^

Theoretical models based on cognitive-behavioral therapy (CBT) have been successfully applied to treat health anxiety, showing evidence of cognitive, affective, and behavioral symptom domains.^19^ People with health anxiety show an attentional focus on threatening health-related information (cognitive). They have a tendency to interpret benign body sensations as dangerous signs of illness, which results in negative emotions (affective). Finally, those exhibiting health anxiety show behavioral avoidance of situations perceived to be health threats (behavioral). Therapies focused on the cognitive-behavioral model have been successfully developed and tested for health anxiety.^5^

CBT has proved to be quite effective for anxiety in general^20^ and for health anxiety in particular (5). Meta-analyses on the efficacy of CBT for health anxiety have shown that CBT reduces health anxiety levels more effectively than waiting list control groups, treatment-as-usual groups, medication groups, and a variety of other therapies.^5,21^ Moreover, the effects of CBT for health anxiety tend to reduce other types of psychological distress, such as depression, and the therapy has shown lasting post-treatment effects in 6- and 12-month follow-up studies.^5^ Therefore, CBT appears to be an appropriate and effective therapeutic approach for the management of health anxiety.

Even though CBT shows excellent efficacy for health anxiety, traditional therapy modalities may be limited during a disease outbreak. Complications such as social distancing, quarantine, shortage of qualified therapists, and delayed-care provisions have made it difficult to access face-to-face therapeutic interventions during the COVID-19 outbreak. While telehealth (e.g., application-based synchronous meetings with health care providers) has become popular, sessions can be costly, and there may be limited providers during times of large-scale mental health needs. Video-based self-administered intervention programs, in turn, can deliver psycho-educational information focused on reducing anxiety through video lessons, reading, and activities accessed via applications, the Internet, or email. Rooted in a CBT framework, video-based interventions have the potential to reduce health-anxiety symptoms in a large population at a low cost. Moreover, video-based interventions may be especially helpful in subpopulations who experience high stigma over mental health problems, and thus may be reluctant to seek therapy.

There has been little research on self-administered video-based programs for health anxiety. Research findings are also difficult to interpret given the diversity in the administration and content of programs. Self-administered video-based CBT-based programs have shown effectiveness for sexual anxiety and pain^22^ and insomnia.^23^ Hedman et al.^24^ examined the efficacy of an Internet-based CBT therapy program for severe health anxiety and found reductions in anxiety and depression among those who participated in the program compared to a control group. However, that program was administered using self-help text-based modules with access to a web-based therapist.^24^ Therefore, more research is needed to examine video-based cognitive-behavioral interventions for health anxiety that do not provide access to a therapist.

Given the dangerous psychological and behavioral consequences of health anxiety, and the tendency for such anxiety to increase dramatically during a public health crisis, it is imperative to find low-cost, large-scale means of managing health anxiety during disease outbreaks. The purpose of the present study was to examine the efficacy of a video-based cognitive-behavioral intervention during a large-scale health crisis, namely the COVID-19 pandemic. We tested whether a video-based intervention, developed using the cognitive-behavioral model, could cause a significant reduction in health anxiety symptoms when compared to a waiting list control condition. Given the success of CBT for health anxiety,^5,21^ and the promising results of CBT video-based treatments,^22,23^ we hypothesized that our cognitive-behavioral video-based intervention would reduce COVID-19 anxiety, health anxiety, anxiety sensitivity, and somatosensory amplification when compared to a randomly assigned control condition.

## Method

### Participants

A convenience sample of college students from Guilan University, in Rasht, Iran, were recruited in March and April of 2020 during the outbreak of COVID-19. This study and its trial protocol were approved by Department of Psychology of Guilan University, Iran. Ethical considerations such as participant satisfaction, data retention and destruction, and informed participation were taken into account in accordance with the Declaration of Helsinki. Additionally, written informed consent was obtained from each participant.

In order to be included in the study, participants were required to: 1) report COVID-19 anxiety symptoms as assessed using the COVID-19 Anxiety Questionnaire (CVAQ) and DSM-5 criteria for illness anxiety disorder^25^; 2) have access to a personal computer with Internet service; 3) be between 18 and 40 years old; and 4) provide written consent. Respondents were excluded from participation if they: 1) reported severe psychological or bodily impairments; 2) reported severe visual and hearing defects; 3) were currently participating in other psychological or physical treatments; 4) missed more than one session; or 5) were reluctant to cooperate.

### Procedure

**Figure 1:**
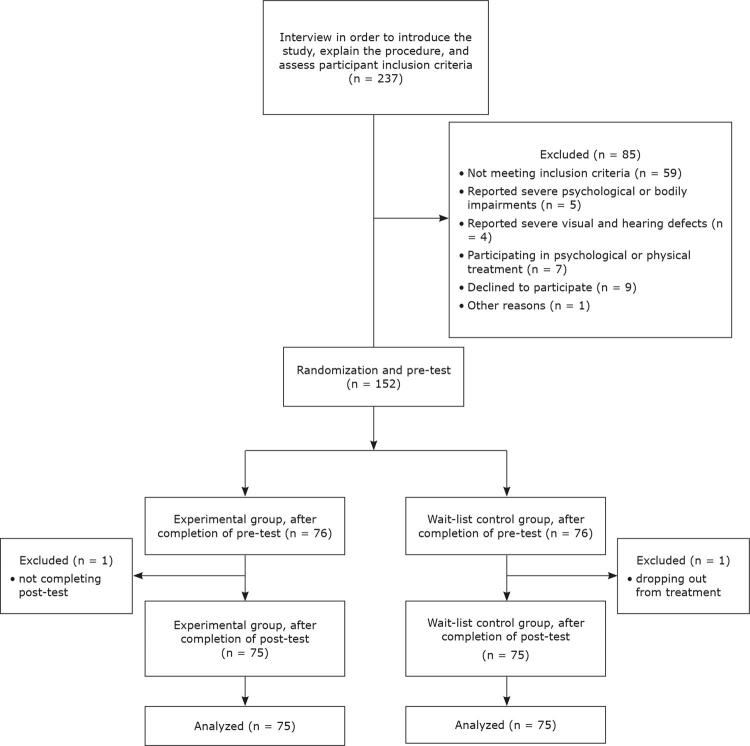
Diagram illustrating participation in pre-test and post-test phases.

Participants were recruited using an online advertisement posted in a college student social network. The advertisement explained that the study examined a video-based cognitive-behavioral intervention for COVID-19 anxiety. Respondents (n = 237) were initially interviewed in order to introduce the study, explain the procedure, and assess participant inclusion criteria. Participants who met the inclusion criteria (n = 152) were randomly assigned to either an experimental or a waiting list control group. After coding each participant with a number, a computer-generated list of random numbers was used to generate the random allocation. Two participants were excluded from the sample following randomization, based on exclusion criteria, resulting in a final sample of 150 participants (Figure 1), of which 77 were women and 73 were men. The mean age of participants was 24.7 years (standard deviation [SD] = 5.4). Also, 127 were undergraduate students and the rest were graduate students (n = 23).

### Design

The present study employed a one-way, randomized, pretest/post-test experimental design to examine the efficacy of an intervention intended to reduce COVID-19 anxiety. Participants were randomly assigned to one of two levels of the independent variable: they either received a video-based, cognitive-behavioral intervention or had their names added to a waiting list and did not receive the intervention during the period of data collection. Prior to manipulation of the independent variable, all participants completed an emailed pre-test that included CVAQ, Short Health Anxiety Inventory (SHAI),^26^ Anxiety Sensitivity Index-3 (ASI-3),^27^ and Somatosensory Amplification Scale (SSAS).^28^ After manipulation of the independent variable, all participants completed an emailed post-test that was identical to the pre-test, with the following additional tests for the cognitive-behavioral intervention group: Experience of Parasocial Interaction Scale (EPSI),^29^ Source Credibility Scale,^30^ a satisfaction item, and an alternative to traditional face-to-face service item. These items were used as a manipulation check to assess the degree to which the participants in the intervention group were engaged with the video-based intervention and judged the source of the information to be credible.

### Measures

#### COVID-19 Anxiety Questionnaire (CVAQ)

To measure COVID-19 anxiety, we adapted items from an existing survey measuring anxiety in the swine flu epidemic (Swine Flu Inventory^31^). The questionnaire consisted of 10 items that covered the respondents’ anxiety related to the COVID-19 pandemic, including concerns about the spread of COVID-19 (e.g., “to what extent do you believe that COVID-19 could become a pandemic in Iran?”); perceived likelihood of contracting the disease (e.g., “how likely is it that you could become infected with COVID-19?”); perceived severity of the disease (e.g., “if you did become infected with COVID-19, to what extent are you concerned that you will be severely ill?”); exposure to information about the disease (e.g., “how much exposure have you had to information about COVID-19?”); and safety behaviors (e.g., “to what extent has the threat of COVID-19 influenced your behaviors, including wearing a mask or using hand sanitizer?”). The questionnaire items are rated on a five-point Likert scale. The lowest total score is 10 and the highest 50. Higher scores are indicative of greater COVID-19 anxiety. Content validity and reliability of the CVAQ have been established.^4^ In the present study, the content validity index (CVI) and content validity ratio (CVR) scores for CVAQ were 0.89 and 0.90, respectively, which indicate good content validity.^32,33^ Additionally, a strong correlation between CVAQ and SHAI (*r* = 0.62; p < 0.01) provided evidence of convergent validity. The reliability of the CVAQ in the present sample was acceptable (Cronbach’s alpha = 0.75).

#### Short Health Anxiety Inventory (SHAI)

The SHAI^26^ is used to measure exaggerated estimates of the likelihood and severity of having an illness. The 18-item SHAI is a shorter version of the 64-item Health Anxiety Inventory (HAI), which measures respondents’ perceived illness likelihood, illness severity, and body vigilance. Each question in SHAI consists of a group of four statements that are scored from 0 to 3. Total scores may range from 0 to 54, with higher scores representing more health anxiety. Salkovskis et al.^26^ reported satisfactory reliability, validity, and sensitivity to treatment for SHAI; convergent, divergent, and predictive validity have been confirmed by Abramowitz, et al.^34^ Previous studies confirmed the capability of SHAI to evaluate health anxiety across samples.^35^ In the present sample, the alpha reliability was 0.81.

#### Anxiety Sensitivity Index-3 (ASI-3)

The ASI-3^27^ assesses concern associated with possible negative consequences of anxiety-related symptoms. The ASI-3 is derived from the Anxiety Sensitivity Index-Revised (ASI-R). The scale consists of 18 items evaluating physical concerns (6 items; e.g., “when my stomach is upset, I worry that I might be seriously ill”), cognitive concerns (6 items; e.g., “when I cannot keep my mind on a task, I worry that I might be going crazy”), and social concerns (6 items; e.g., “I worry that other people will notice my anxiety”) related to anxiety. Items are rated on a five-point Likert scale ranging from 0 (very little) to 4 (very much) and summed to create a total score (0-72). The ASI-3 has shown satisfactory convergent validity, divergent validity, and reliability.^27^ Petrocchi et al.^36^ confirmed the three-factor structure of the ASI-3. They reported Cronbach’s alphas of 0.87, 0.83, 0.81 and 0.90 for physical concerns, social concerns, cognitive concerns, and total index, respectively. In the present study, the ASI-3 showed good internal consistency (α = 0.83).

#### Somatosensory Amplification Scale (SSAS)

The SSAS is a 10-item self-assessment instrument with response options rated on a scale from 1 to 5 and the total score ranging from 10 to 50. The SSAS evaluates the tendency to experience normal somatosensory sensations as intense. Validity, test-retest reliability (*r* = 0.79; p < .0001), and internal consistency (α = 0.82) of the SSAS were shown to be adequate by Barsky et al.^28^ Previous studies confirm suitable psychometric properties of SSAS in Japanese,^37^ Turkish,^38^ and Iranian^39^ populations. In this study, Cronbach’s alpha was 0.81.

#### Experience of Parasocial Interaction Scale (EPSI)

Hartmann & Goldhoorn^29^ developed the EPSI to examine viewers’ parasocial bond experience with a TV performer. After viewing the video clips, participants in the cognitive-behavioral intervention group responded to the EPSI. The EPSI is a single-factor, six-item questionnaire scored on a seven-point Likert scale ranging from 1 (do not agree at all) to 7 (totally agree). Items cover mutual awareness, attention, and adjustment to the performer featured in the video (e.g., “while watching the clip, I had the feeling that [the performer] knew I paid attention to him/her”). Higher scores represent a more intense parasocial experience with the video clip performer. Findings of Hartmann & Goldhoorn^29^ and Shabahang et al.^40^ confirm appropriate psychometric properties of the ESPI. Our sample showed good Cronbach’s alpha for the scale (α = 0.86).

#### Source Credibility Scale

The Source Credibility Scale is a 15-item semantic differential scale to measure perceived attractiveness (attractiveness, chicness, and sexiness), trustworthiness (confidence and acceptance), and expertise (expertness) of a performer. This scale was administered only to the group receiving the video-based intervention. Items of the Source Credibility Scale are scored on a seven-point scale. Ohanian^30^ has established the scale’s validity and reliability. In this study, alpha reliability was 0.87.

#### Satisfaction item

Satisfaction with the video-based cognitive-behavioral intervention was measured using the following question: “How satisfied were you with the intervention?” The respondent rated satisfaction on a 10-point Likert scale (1 = from not at all; to 10 = very much).

#### Alternative to traditional face-to-face service item

The participants were also asked to respond to the following question: “Do you experience the video-based cognitive-behavioral intervention as a beneficial alternative to traditional face-to-face service?” Participants answered the question with yes or no.

## Video-based cognitive-behavioral intervention

Participants randomly assigned to the video-based cognitive-behavioral intervention group received a self-help package, including nine video clips (153 minutes of video in total) and a 25-page online booklet. Participants were instructed to first watch a video clip (15-20 minutes each) and then read the corresponding pages of the online booklet (2-3 pages each) for 3 days of each week over the course of 3 consecutive weeks. In keeping with best practices for maximizing persuasion,^41^ we sought to increase credibility of information in the video by citing scientific sources. Additionally, we kept videos short and goal-oriented to lower the intrinsic cognitive load of the participants.^42^ Finally, we employed attention cueing^43^ by highlighting important concepts using on-screen symbols and text.

The intervention’s content combined cognitive-behavioral, social, and educational strategies to reduce anxiety. The script for the video and the content of the booklet were based on previous CBT protocols for health anxiety. The intervention covers the following components designed to lower health anxiety: positive appraisal, non-catastrophic beliefs, less-threatening explanations, reduction of false safety-seeking behaviors,^44^ challenge of automatic thoughts^45^; introduction of alternative explanations^46^; shared understanding^47^; reduction of biased intrusive images^1^; examination of attention and bodily hypervigilance, amplification of symptoms, and coping strategies for illness anxiety^48^; mindfulness training^49^; and case illustration.^50^

## Statistical analysis

This study was a randomized controlled trial comparing an intervention group and a waiting list control group to evaluate the efficacy of the intervention. The data obtained were analyzed using the Statistical Package for the Social Sciences (SPSS) version 24. Multivariate analysis of covariance (MANCOVA) was used to investigate pre- vs. post-treatment differences.

## Ethics statement

The present study was conducted in coordination with the Department of Psychology of Guilan University, Iran. All ethical considerations such as personal satisfaction, data retention and destruction, and informed participation were taken into account in accordance with the Declaration of Helsinki.

## Results

Table 1 presents mean and SD scores obtained on COVID-19 anxiety, health anxiety, anxiety sensitivity, and somatosensory amplification in the video-based cognitive-behavioral intervention and waiting list control groups. Kolmogorov-Smirnov test results suggested that the study variables followed a normal distribution in our population.

**Table 1:** Descriptive indices of the study variables in experimental and waiting list control groups

Variable/group	Mean	SD	K-S	p
COVID-19 anxiety				
Pre-test				
Intervention group	37.68	1.66	0.079	0.052
Waiting list control group	37.62	3.21	0.090	0.072
Post-test				
Intervention group	30.61	4.01	0.078	0.142
Waiting list control group	37.25	3.32	0.145	0.082
Health anxiety				
Pre-test				
Intervention group	40.92	9.24	0.059	0.068
Waiting list control group	40.83	7.97	0.089	0.077
Post-test				
Intervention group	30.21	10.65	0.071	0.063
Waiting list control group	40.52	8.31	0.087	0.099
Anxiety sensitivity				
Pre-test				
Intervention group	48.64	6.56	0.091	0.097
Waiting list control group	48.58	8.74	0.098	0.067
Post-test				
Intervention group	40.73	8.19	0.076	0.055
Waiting list control group	48.96	7.84	0.089	0.118
Somatosensory amplification				
Pre-test				
Intervention group	35.74	10.15	0.083	0.056
Waiting list control group	35.58	10.36	0.094	0.070
Post-test				
Intervention group	25.71	10.36	0.093	0.103
Waiting list control group	35.14	10.42	0.076	0.057

COVID-19 = coronavirus disease 2019; KS = Kolmogorov-Smirnov; SD = standard deviation.

Findings of Levene’s test for checking the assumption of equal variances confirmed that the variances of COVID-19 anxiety (F_1,148 _= 0.23, p = 0.636 > 0.05), health anxiety (F_1,148 _= .30, p = .580 > .05), anxiety sensitivity (F_1,148 _= 0.36, p = 0.462 > 0.05), and somatosensory amplification (F_1,148 _= 0.72, p = 0.395 > 0.05) were similar between the two groups. The results of Box’s M indicated that the observed covariance matrices of the dependent variables were similar across groups (Box’s M = 37.83, F = 1.83, p = 0.064 > 0.05). Bartlett’s test for sphericity confirmed the relatedness between COVID-19 anxiety, health anxiety, anxiety sensitivity, and somatosensory amplification (χ^2^ = 287.34, degrees of freedom [df] = 9, p < 0.01). Additionally, the assumption of homogeneity of regression slopes was tested. Homogeneity of regression coefficients was investigated through the interaction of dependent and independent variables in pre-test and post-test. The interaction of these pre-tests and post-tests with the independent variable was not significant, indicating that the assumption of homogeneity of the regression slope was established.

The results of Table 2 show the effect of the independent variable on the dependent variables. The intervention and waiting list control groups showed a significant difference in at least one of the variables of COVID-19 anxiety, health anxiety, anxiety sensitivity, and somatosensory amplification. Sixty-three percent of total variances of the experimental and waiting list control groups were due to the independent variable. The statistical power of the test was also equal to 1, indicating adequacy of the sample size. However, in order to determine which domains were significant, univariate analysis of covariance was used in the MANCOVA, the results of which are reported in Table 3.

**Table 2:** Results of the multivariate analysis of covariance on mean post-test scores

Test	Value	F	p	Effect size (η_p_^2^)
Pillai’s effect	0.63	60.15	0.001	0.63
Wilks lambda	0.37	60.15	0.001	0.63
Hotelling’s trace	1.71	60.15	0.001	0.63
Roy’s largest root	1.71	60.15	0.001	0.63

**Table 3:** Results of the univariate analysis of covariance on mean post-test scores of dependent variables in both experimental and waiting list control groups

Variable	SS	DF	MS	F	p	Effect size (η_p_^2^)
COVID-19 anxiety	1,655.98	1	1,655.98	139.22	0.001	0.49
Health anxiety	4,034.43	1	4,034.43	42.97	0.001	0.23
Anxiety sensitivity	2,543.03	1	2,543.03	40.47	0.001	0.22
Somatosensory amplification	3,384.98	1	3,384.98	38.74	0.001	0.21

DF = degrees of freedom; MS = mean square; SS = sum of squares.

There was a significant difference between the intervention and control groups in COVID-19 anxiety (*F* = 139.22; p < 0.01), health anxiety (*F* = 42.97; p < 0.01), anxiety sensitivity (*F* = 40.47; p < 0.01), and somatosensory amplification (*F* = 38.74; p < 0.01). Cohen^51^ suggested that small, medium, and large effect sizes are 0.2, 0.5, and 0.8, respectively. Small to medium effect sizes were obtained for COVID-19 anxiety, health anxiety, anxiety sensitivity, and somatosensory amplification. Our findings suggest that the video-based cognitive-behavioral intervention was slightly to moderately effective in lowering COVID-19 anxiety, health anxiety, anxiety sensitivity, and somatosensory amplification of individuals with high levels of COVID-19 anxiety.

Additionally, participants in the intervention group reported a high experience of parasocial interaction (mean = 32.10; SD = 4.56), source credibility (mean = 81.81; SD = 10.14), and satisfaction (mean = 7.69; at least = 1.12) with the video-based cognitive-behavioral intervention. A majority of participants (80%) evaluated the intervention as a beneficial alternative to traditional face-to-face therapeutic interventions.

## Discussion

To the best of our knowledge, this study was the first to investigate the effectiveness of a video-based psychological intervention for COVID-19 anxiety. Our randomized controlled experimental design showed that our video-based cognitive-behavioral intervention significantly reduced COVID-19 anxiety, health anxiety, anxiety sensitivity, and somatosensory amplification in individuals with high levels of COVID-19 anxiety. These results suggest that video-based cognitive-behavioral interventions might serve as viable alternatives to traditional face-to-face therapeutic interventions for health anxiety during large-scale public health crises such as the COVID-19 pandemic.

Similar to previous epidemic diseases, COVID-19 has caused members of many communities to feel anxious and frustrated. Cognitive-behavioral interventions have proved quite successful in anxiety reduction,^20^ especially health anxiety reduction.^5^ However, certain aspects of pandemics (e.g., social distancing, quarantines, shortage of experts, and delayed care provisions) may make it impossible to administer face-to-face therapeutic interventions at a large scale. Video-based therapeutic tools can offer opportunities to provide effective psychological interventions for COVID-19 anxiety to large populations. Our findings support the applicability of video-based cognitive-behavioral intervention on COVID-19 anxiety. In other words, the results of this study suggested that a self-help video-based cognitive-behavioral intervention was an efficacious means of relieving COVID-19 anxiety, health anxiety, anxiety sensitivity, and somatosensory amplification.

Symptoms of health anxiety,^34,52^ illness cognition,^53^ the cognitive-behavioral model of health anxiety^19^ and reports of health anxiety from previous pandemics^31^ suggest that individuals with high levels of COVID-19 anxiety are likely to overestimate the probability of having COVID-19, show excessive preoccupation about COVID19, have catastrophic beliefs, have difficulty controlling feelings of worry, show compulsive checking of bodily signs, and misinterpret normal bodily variations to be signs of illness. Those suffering from COVID-19 anxiety are also likely to perform unreasonable health-related behaviors, such as reassurance seeking and researching illness and treatments. Our results showed that a video-based cognitive-behavioral intervention was able to help participants to identify their health anxiety worries and beliefs, find alternative, less-threatening explanations, create positive appraisal about their health, reduce their biased intrusive images, respond less intensely to body sensations, and cope with illness anxiety.

Additionally, participants considered the video-based intervention to be informative and interesting. The participants showed strong parasocial bonds with the TV performer. They also evaluated the intervention as a valid, satisfactory, and appropriate alternative to face-to-face intervention. These findings indicate that participants showed strong engagement with the video and associated text, which contributed to the experimental realism of the intervention.

We regard the findings of the present study to be of high relevance from a clinical perspective. First, COVID-19 has led to widespread health anxiety that may carry long-term physical and psychological consequences. Second, a cognitive-behavioral model can be used to help individuals interpret and understand COVID-19 anxiety. Third, video-based cognitive-behavioral intervention is a low-cost, feasible and effective service that can lead to significant reduction in COVID-19 anxiety, health anxiety, anxiety sensitivity, and somatosensory amplification.

Our study had several important limitations. Our initial selection of participants was opportunistic. Participants were students, i.e., they do not represent the diversity found in broader community populations. The measurements were not masked completely, and therefore may have been subject to participant demand characteristics. Only self-report measurements were used, which made it difficult to assess the degree to which reported reductions in anxiety may have affected behavior. Not measuring adherence metrics such as video clips watched, pages of the booklet read, and days used was another limitation of our study. Our effect sizes were relatively low. Since the effects of CBT demonstrate a dose-response relationship,^21^ we can guess that more than nine sessions could have produced larger effects. It would also be desirable to have follow-up measures to examine for how long reductions in anxiety were maintained. Finally, our intervention was tested only against a wait-listed control group. While this design showed that the intervention was effective, we cannot be sure of which aspects of the intervention were the most important or whether an alternative intervention (e.g., non-CBT based) could have been equally effective. However, previous research^5^ has compared CBT to a variety of control conditions (e.g., waiting list, other psychotherapies, and medication) and found it to be highly effective for health anxiety, even at > 1 year post-therapy.

## Conclusion

Video-based interventions give individuals the opportunity to learn cognitive-behavioral strategies for overcoming COVID-19 anxiety at home, during times that are best suited for their schedules. Given the high prevalence of health anxiety and barriers to intervention during large-scale public health crises, our results show that video-based cognitive-behavioral intervention could benefit individuals and societies during times of widespread panic.

**Figure 2:**
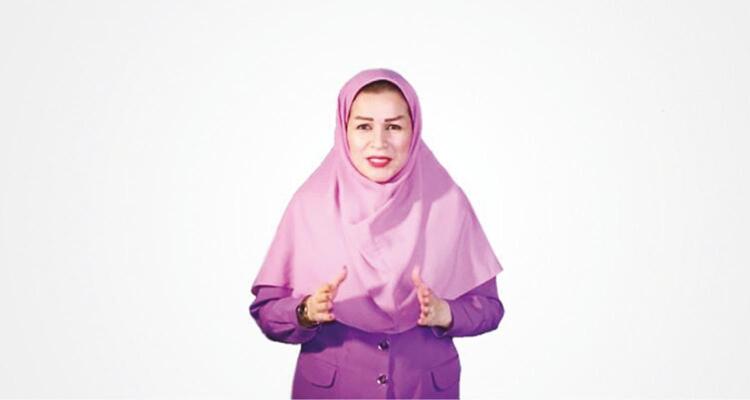
Illustration extracted from the video-based cognitive-behavioral intervention.
